# Impact of Prior Myocardial Infarction on Outcomes Following Multiple Arterial Coronary Bypass Grafting: A Propensity-Matched Analysis †

**DOI:** 10.3390/jcdd13060272

**Published:** 2026-06-16

**Authors:** Albaraa Al-Holy, Nandor Marczin, Sunil K. Bhudia, Shahzad G. Raja

**Affiliations:** 1 Department of Cardiac Surgery, Harefield Hospital, London UB9 6JH, UK; albaraa.alholy1@nhs.net (A.A.-H.);; 2 Department of Anaesthesia and Intensive Care, Harefield Hospital, London UB9 6JH, UK

**Keywords:** arterial grafting, coronary artery bypass, myocardial infarction, propensity score, survival analysis

## Abstract

**Background:** Multiple arterial grafting (MAG) is associated with superior long-term outcomes in coronary artery bypass grafting (CABG). The influence of prior myocardial infarction (MI) on outcomes following MAG remains uncertain. This study evaluates in-hospital outcomes and long-term survival of MAG in patients with and without previous MI. **Methods:** A retrospective single-center observational analysis of 2468 patients undergoing MAG was performed. Propensity score matching yielded 911 pairs based on preoperative variables. Kaplan–Meier survival analysis and Cox regression were used to assess long-term survival and predictors of mortality. **Results:** In the unmatched cohort, patients with prior MI had significantly higher rates of diabetes (30.6% vs. 23.9%, *p* < 0.001), smoking history (*p* < 0.001), and impaired left ventricular function (fair/poor LVEF: 32.4% vs. 11.1%, *p* < 0.001), along with higher logistic EuroSCORE (3.81 vs. 3.11, *p* < 0.001). After matching, baseline characteristics were balanced. In-hospital outcomes, including 30-day mortality (1.5% vs. 1.9%, *p* = 0.587), stroke, reoperation, and renal complications, were similar. Long-term survival at 10, 15, and 20 years was comparable (log-rank *p* = 0.814). Multivariate Cox regression identified age (HR 1.065, *p* < 0.001), NYHA class, diabetes (HR 0.779, *p* = 0.008), and off-pump CABG (HR 1.444, *p* < 0.001) as independent predictors of mortality. Prior MI was not associated with increased long-term mortality (HR 0.872, *p* = 0.105). **Conclusions:** Despite worse baseline risk profiles, patients with prior MI undergoing MAG had equivalent in-hospital outcomes and long-term survival. MAG remains a robust revascularization strategy irrespective of MI history, supporting its broader use in CABG. These findings should be interpreted in the context of a single-center experience from a high-volume arterial grafting program.

## 1. Introduction

Patients with a history of myocardial infarction (MI) constitute a clinically important and increasingly prevalent subgroup among those undergoing coronary artery bypass grafting (CABG) [1,2]. Prior MI is associated with adverse ventricular remodeling, microvascular dysfunction, and increased susceptibility to recurrent ischemic events, all of which contribute to elevated perioperative risk and diminished long-term survival [3,4]. As a result, this population has traditionally been considered higher risk, with concerns that myocardial scarring, impaired contractile reserve, and diffuse coronary disease may attenuate the benefits of surgical revascularization [5,6].

Multiple arterial grafting (MAG) has emerged as a revascularization strategy associated with superior long-term patency, reduced graft failure, and improved survival compared with conventional single arterial grafting [7,8]. Arterial conduits, particularly the internal thoracic and radial arteries, demonstrate enhanced resistance to atherosclerosis and provide more durable myocardial perfusion over time [9,10]. However, the extent to which these advantages translate into improved outcomes in patients with prior MI remains uncertain. These patients often present with more extensive coronary disease, impaired left ventricular function, and a greater burden of comorbidities, raising the possibility that the benefits of MAG may be attenuated by competing risks [11].

Despite the established prognostic implications of prior MI, there is a paucity of data specifically evaluating its impact on outcomes following MAG. Understanding whether previous infarction influences perioperative morbidity or long-term survival in this context is essential for optimizing conduit strategy and refining patient selection. This study therefore aimed to assess the effect of prior MI on in-hospital outcomes and long-term survival in patients undergoing MAG, using a large single-center cohort and robust propensity-matched methodology. Secondary objectives included identifying independent predictors of long-term mortality and determining whether prior MI independently influences survival after MAG.

## 2. Methods

### 2.1. Study Design and Population

This retrospective observational study included 2468 consecutive patients who underwent multiple arterial coronary artery bypass grafting (MAG) at a high-volume tertiary cardiac surgery center between January 1996 and September 2023. All patients received at least two arterial conduits, most commonly combinations of left and right internal thoracic arteries and radial artery grafts. Patients undergoing concomitant cardiac procedures (e.g., valve surgery, aortic surgery) were excluded to ensure a homogeneous cohort focused exclusively on isolated coronary revascularization.

Patients were stratified into two groups based on the presence or absence of documented prior myocardial infarction. Prior myocardial infarction was defined using a combination of routinely collected clinical data, including documented medical history, electrocardiographic evidence of prior infarction, elevation of cardiac biomarkers at the time of the index event or prior admission records, angiographic findings consistent with previous infarction, and imaging evidence where available (e.g., echocardiography or other institutional imaging modalities), in accordance with standard institutional diagnostic practice over the study period. However, detailed characterization of infarct characteristics—including timing (recent versus remote myocardial infarction), infarct territory, infarct size, infarct type, viability assessment, and scar burden—was not consistently available across the full study period (1996–2023) and therefore could not be analysed reliably.

### 2.2. Data Collection

Data were obtained from a prospectively maintained institutional database with comprehensive capture of demographic, clinical, angiographic, operative, and postoperative variables. Preoperative variables included age, sex, comorbidities, New York Heart Association (NYHA) class, left ventricular ejection fraction (LVEF), extent of coronary artery disease, and logistic EuroSCORE. Operative variables included number of grafts, conduit configuration, and use of off-pump CABG (OPCAB). Postoperative outcomes included reoperation for bleeding, stroke/transient ischaemic attack (TIA), deep sternal wound infection (DSWI), renal replacement therapy (RRT), and 30-day mortality. Long-term survival was obtained through national mortality registries and hospital records.

### 2.3. Outcomes

The primary endpoint was long-term all-cause mortality. Secondary endpoints included in-hospital complications and 30-day mortality. Survival time was calculated from the date of surgery to death or last follow-up.

### 2.4. Statistical Analysis

Continuous variables were compared using independent-samples *t*-tests or Mann–Whitney U tests, depending on distribution. Categorical variables were compared using χ^2^ or Fisher’s exact tests. To complement *p* values, effect estimates with 95% confidence intervals (95% CI) were calculated for all in-hospital outcomes. Risk ratios (RR) were used for binary variables, including 30-day mortality and postoperative complications. Kaplan–Meier survival curves were generated for unmatched and matched cohorts, with differences assessed using the log-rank test. Cox proportional hazards regression was used to identify predictors of long-term all-cause mortality, with death coded as the event of interest and survival time calculated from the date of surgery to death or last follow-up. Variables with *p* < 0.10 on univariable analysis were entered into the multivariable model using a clinically guided approach to minimize model overfitting relative to the number of observed mortality events. Reference categories for categorical covariates were predefined prior to analysis. Proportional hazards assumptions were assessed using Schoenfeld residual testing and inspection of log-minus-log survival plots, with no significant violations identified.

To minimize confounding and balance baseline characteristics between groups, propensity score matching was performed using 1:1 nearest-neighbor matching without replacement. All preoperative variables listed in Table 1 were included in the logistic regression model. Although logistic EuroSCORE was included to capture overall operative risk, we acknowledge that it partially incorporates several individual covariates included in the model, and therefore may introduce a degree of conceptual overlap and potential overadjustment. A caliper width of 0.10 of the standard deviation of the logit of the propensity score was applied. The final matched cohort comprised 911 pairs (n = 1822), with excellent covariate balance across all variables. Balance between groups was further assessed using standardized mean differences (SMDs), with values < 0.10 considered indicative of negligible imbalance. Outcome analyses in the propensity-matched cohort were performed using regression models incorporating robust standard errors to account for clustering within matched pairs.

Statistical significance was defined as *p* < 0.05. All statistical analyses were performed using IBM SPSS Statistics (Version 29.0; IBM Corp., Armonk, NY, USA).

## 3. Results

### 3.1. Preoperative Demographics

Preoperative characteristics are summarised in Table 1. In the unmatched cohort, patients with prior MI demonstrated a significantly different clinical profile compared with those without prior MI. They were slightly younger (60.5 ± 9.5 vs. 61.5 ± 9.4 years, *p* = 0.013; SMD = 0.10) but had a higher burden of adverse cardiovascular risk factors, including a greater prevalence of diabetes (30.6% vs. 23.9%, *p* < 0.001; SMD = 0.15) and a more pronounced smoking history (*p* < 0.001; SMD = 0.19), with higher proportions of both current and ex-smokers. Functional status also differed significantly, with worse NYHA class distribution in the prior MI group (*p* = 0.038; SMD = 0.13). Most notably, patients with prior MI had substantially impaired left ventricular function, with markedly lower rates of preserved LVEF and higher proportions of moderate and severe dysfunction (*p* < 0.001; SMD = 0.56), representing the largest baseline imbalance. In keeping with this higher-risk profile, logistic EuroSCORE was also significantly elevated in the prior MI group (3.81 ± 4.9 vs. 3.11 ± 3.8, *p* < 0.001; SMD = 0.16).

Following propensity score matching, all baseline characteristics were well balanced between groups. No statistically significant differences remained, and standardized mean differences were reduced to <0.10 across all variables, including LVEF (SMD = 0.01) and logistic EuroSCORE (SMD = 0.06), indicating minimal residual imbalance and ensuring appropriate comparability for subsequent outcome analyses.

### 3.2. Intraoperative Characteristics

Operative details are summarised in Table 2. In the unmatched cohort, there were no statistically significant differences between patients with and without prior MI across key intraoperative variables. The proportion of OPCAB procedures was comparable (50.1% vs. 53.3%, *p* = 0.114), as was the mean number of grafts performed (3.30 ± 0.55 vs. 3.28 ± 0.54, *p* = 0.588). Similarly, cardiopulmonary bypass (CPB) time (83 [62–86] vs. 84 [65–88] minutes, *p* = 0.812) and aortic cross-clamp time (50 [37–60] vs. 50 [38–61] minutes, *p* = 0.912) were equivalent between groups. The index of completeness of revascularization (ICOR) showed a borderline difference, being slightly lower in the prior MI group (1.14 ± 0.10 vs. 1.18 ± 0.12, *p* = 0.055), although this did not reach statistical significance. Grafting of the left anterior descending (LAD) artery was performed in all patients in both groups (100%).

These findings remained consistent following propensity score matching. In the matched cohort, no significant differences were observed in OPCAB use (51.0% vs. 51.8%, *p* = 0.743), number of grafts (3.29 ± 0.55 vs. 3.31 ± 0.56, *p* = 0.475), CPB time (83 [61–90] vs. 83 [63–91] minutes, *p* = 0.824), or aortic cross-clamp time (50 [36–61] vs. 51 [37–64] minutes, *p* = 0.854). ICOR was also similar between groups after matching (1.14 ± 0.13 vs. 1.17 ± 0.14, *p* = 0.674).

Overall, intraoperative management, including operative technique, grafting strategy, and completeness of revascularization, was well matched between groups, indicating that differences in outcomes are unlikely to be attributable to procedural factors.

### 3.3. In-Hospital Outcomes

Postoperative outcomes are summarised in Table 3. In the unmatched cohort, effect estimates for in-hospital outcomes were generally close to unity, with wide confidence intervals and no clear evidence of clinically meaningful differences between patients with and without prior myocardial infarction. Reoperation rates were similar between groups (4.3% vs. 3.7%; RR 0.86, 95% CI 0.58–1.27; *p* = 0.840), as were tracheostomy requirements (0.9% vs. 1.2%; RR 1.38, 95% CI 0.64–2.97; *p* = 0.405) and cerebrovascular events including transient ischaemic attack or stroke (1.9% vs. 2.1%; RR 1.14, 95% CI 0.65–1.99; *p* = 0.646). The incidence of deep sternal wound infection was identical between groups (1.3% vs. 1.3%; RR 0.99, 95% CI 0.50–1.96; *p* = 1.000), while renal replacement therapy was slightly higher in the prior MI group but with imprecise estimates (1.7% vs. 2.5%; RR 1.49, 95% CI 0.87–2.56; *p* = 0.149). Thirty-day mortality was numerically higher in patients with prior MI (1.5% vs. 2.6%), corresponding to a borderline and imprecise increase in risk (RR 1.72, 95% CI 0.98–3.02; *p* = 0.056).

Following propensity score matching, all in-hospital outcomes remained well balanced between groups, with effect estimates consistently close to unity. Reoperation rates (4.1% vs. 3.8%; RR 0.94, 95% CI 0.60–1.48; *p* = 0.810), tracheostomy (0.9% vs. 1.2%; RR 1.38, 95% CI 0.56–3.41; *p* = 0.489), and cerebrovascular events (1.9% vs. 2.0%; RR 1.06, 95% CI 0.55–2.04; *p* = 0.864) were comparable between groups. Similarly, deep sternal wound infection (0.4% vs. 0.2%; RR 0.50, 95% CI 0.09–2.72; *p* = 0.687) and renal replacement therapy (1.5% in both groups; RR 1.00, 95% CI 0.48–2.09; *p* = 1.000) showed no evidence of differential risk. Thirty-day mortality remained low and comparable after matching (1.5% vs. 1.9%; RR 1.21, 95% CI 0.60–2.44; *p* = 0.587).

Overall, across both unmatched and propensity-matched analyses, effect estimates for early postoperative outcomes were close to unity with overlapping confidence intervals, indicating no clinically meaningful difference in short-term morbidity or mortality between patients with and without prior myocardial infarction undergoing multiple arterial grafting.

### 3.4. Long-Term Survival

Kaplan–Meier survival analysis demonstrated comparable long-term outcomes between patients with and without prior MI. Mean follow-up duration was similar between groups in both the unmatched cohort (12.0 ± 7.5 vs. 11.5 ± 7.3 years, *p* = 0.092; SMD = 0.07) and the matched cohort (11.7 ± 7.5 vs. 11.6 ± 7.3 years, *p* = 0.791; SMD = 0.01), indicating balanced and adequate longitudinal assessment.

**Figure 1:**
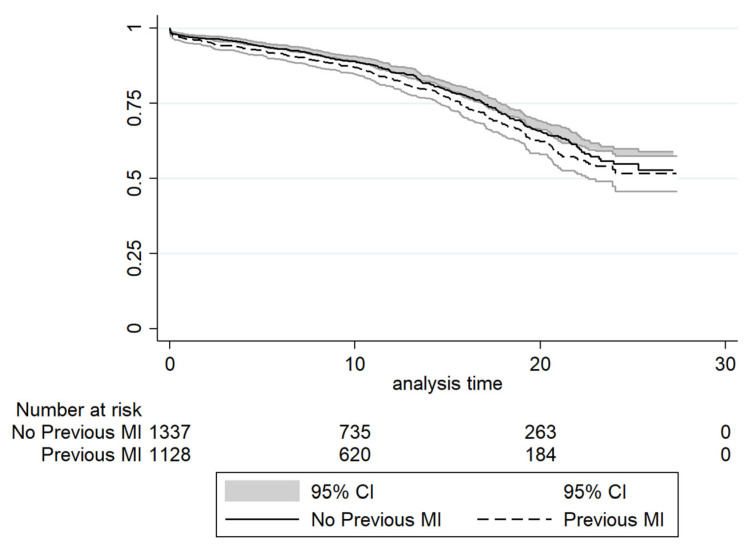
Kaplan–Meier survival analysis of unmatched cohort.

In the unmatched cohort (Table 4), survival estimates were consistently slightly higher in patients without prior MI, although differences were small and not statistically significant (log-rank *p* = 0.1057). One-year survival was 97.1% in patients without prior MI compared to 96.3% in those with prior MI. This pattern persisted over time, with survival at 5 years (93.9% vs. 92.7%), 10 years (88.9% vs. 87.0%), 15 years (79.2% vs. 77.1%), 20 years (65.8% vs. 62.3%), and 25 years (54.9% vs. 51.7%). Despite a numerical trend toward slightly lower survival in the prior MI group, confidence intervals overlapped at all time points, and no statistically significant difference was observed. These findings are illustrated in Figure 1, which shows closely aligned Kaplan–Meier curves throughout the follow-up period.

**Figure 2:**
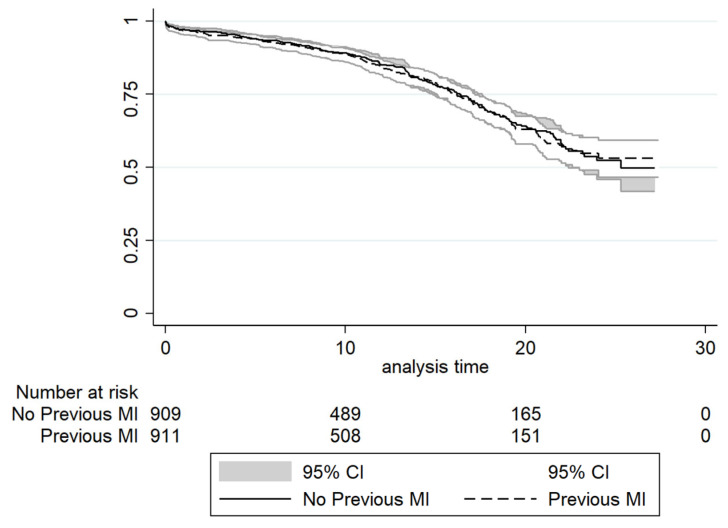
Kaplan–Meier survival analysis of matched cohort.

In the propensity-matched cohort (Table 5), survival curves were even more closely aligned, with virtually identical outcomes between groups (log-rank *p* = 0.8142). One-year survival was 97.1% in patients without prior MI and 96.7% in those with prior MI, while 5-year survival was identical at 93.9% in both groups. Long-term survival remained highly comparable at 10 years (89.1% vs. 88.5%), 15 years (77.9% vs. 78.8%), 20 years (64.2% vs. 63.0%), and 25 years (52.4% vs. 53.2%). The Kaplan–Meier curves (Figure 2) demonstrate near-complete overlap across the entire duration of follow-up, further supporting the absence of any meaningful difference in survival.

Overall, these findings indicate that a history of prior MI does not adversely impact long-term survival following MAG, with consistent results observed in both unmatched and propensity-matched analyses over extended follow-up.

### 3.5. Predictors of Long-Term Mortality

A total of 1149 deaths occurred during follow-up and were included in the Cox regression analysis. Univariable and multivariable Cox proportional hazards analyses are presented in Table 6. Increasing age, advanced NYHA class, diabetes, and OPCAB were identified as independent predictors of long-term mortality. Prior MI was not independently associated with increased mortality (HR 0.872, *p* = 0.105), suggesting that once patients are appropriately selected and revascularised with MAG, the adverse prognostic implications of previous infarction may be mitigated.

## 4. Discussion

This large, propensity-matched analysis demonstrates that prior myocardial infarction does not adversely influence in-hospital outcomes or long-term survival in patients undergoing multiple arterial grafting. Despite presenting with a higher baseline risk profile—including more diabetes, smoking, impaired LVEF, and higher EuroSCORE—patients with prior MI achieved outcomes equivalent to those without previous infarction. These findings suggest that MAG provides durable and effective revascularization irrespective of MI history.

Prior MI is a well-established risk factor for recurrent ischaemia, heart failure progression, and long-term mortality [12]. Comparative studies of CABG versus PCI in patients with previous MI have consistently shown superior protection against recurrent MI and repeat revascularization with surgical revascularization [13]. The pooled analysis of BEST, PRECOMBAT, and SYNTAX trials demonstrated that CABG significantly reduced the composite of death, MI, and stroke compared with PCI in patients with prior MI, driven primarily by a marked reduction in recurrent infarction [14].

Our findings extend this evidence by demonstrating that within the CABG population, the use of multiple arterial conduits provides durable long-term outcomes even in patients with prior MI. The absence of a survival difference between patients with and without prior infarction is consistent with the established long-term durability of arterial grafts, which have been shown in previous studies to exhibit superior patency and resistance to atherosclerosis compared with venous conduits [7,11].

Patients with prior MI often have regions of hibernating or scarred myocardium, impaired microvascular reserve, and increased susceptibility to ischemia [15]. Arterial grafts, with their superior long-term patency and endothelial function, may provide more stable perfusion to jeopardized territories, reducing the risk of late graft failure and recurrent ischemia. The durability of arterial conduits may be particularly advantageous in patients with impaired ventricular function, who rely heavily on sustained graft patency for long-term myocardial support [7,16,17].

Consistent with prior literature, age, NYHA class, diabetes, and OPCAB emerged as independent predictors of long-term mortality [18,19,20]. These findings highlight that patient-level factors and operative characteristics exert a stronger influence on long-term prognosis than MI history or conduit strategy. The lack of association between prior MI and mortality after adjustment suggests that within this selected MAG cohort, previous infarction was not independently associated with worse long-term survival.

This study has several notable strengths that enhance the reliability and interpretability of its findings. The large cohort of 2468 patients undergoing multiple arterial grafting (MAG) provides substantial statistical power and allows meaningful subgroup comparisons, including a robust propensity-matched analysis. The extended follow-up period—spanning more than two decades—offers valuable insight into the long-term durability of arterial revascularization in patients with and without prior myocardial infarction. The use of a prospectively maintained institutional database ensures high data completeness and accuracy, while the application of rigorous propensity score matching effectively balances baseline characteristics and reduces selection bias. By focusing exclusively on MAG, the study isolates the performance of a revascularization strategy increasingly recognized for its superior long-term patency and survival benefits, thereby contributing important evidence to an area where data remain limited. The integration of multivariable Cox regression further strengthens internal validity by identifying independent predictors of mortality and clarifying the relative prognostic contribution of prior MI.

Despite these strengths, several limitations warrant consideration. The retrospective observational design introduces the possibility of unmeasured confounding, even after propensity matching and multivariable adjustment. In addition, the absence of consistently available detailed infarct characterization (including infarct timing, territory, size, and viability or scar assessment) may introduce unmeasured heterogeneity within the prior MI cohort, which should be considered when interpreting the observed findings. Temporal heterogeneity is another important limitation: the 27-year study period encompasses substantial evolution in surgical techniques, conduit harvesting methods, perioperative care, and secondary prevention strategies, any of which may influence outcomes. Echocardiographic follow-up was not routinely available, preventing assessment of postoperative ventricular recovery, graft-related perfusion changes, or the interaction between myocardial viability and conduit strategy. The study also lacks detailed information on infarct size, location, and timing, which may influence both operative risk and long-term prognosis. As a single-center analysis conducted in a high-volume arterial grafting program with extensive expertise in MAG, the findings may not be fully generalizable to centers with different case-mix, surgical experience, or conduit-use patterns. Finally, given the absence of cause-specific mortality, recurrent ischemic events, graft patency imaging, and postoperative functional assessments, mechanistic explanations for the observed long-term survival patterns remain speculative.

The findings of this study have important implications for clinical practice and health-system decision-making. Demonstrating that prior myocardial infarction does not adversely affect perioperative outcomes or long-term survival following MAG suggests that this revascularization strategy is broadly applicable across a wide spectrum of patients, including those traditionally considered higher risk. Although the study was conducted at a high-volume center with extensive experience in arterial grafting, the consistency of outcomes after propensity matching indicates that the benefits of MAG are not confined to a narrow, low-risk subset. Instead, they appear to extend to patients with more complex coronary disease and a history of myocardial injury.

For clinicians, these results support a more inclusive approach to conduit selection. Surgeons may be reassured that prior MI—often associated with impaired ventricular function, microvascular dysfunction, and increased comorbidity burden—should not be viewed as a deterrent to offering MAG. Rather, the durability of arterial conduits may be particularly advantageous in this population, who rely heavily on sustained graft patency to maintain myocardial perfusion and prevent late ischemic events. The absence of excess perioperative morbidity further reinforces the safety of MAG in patients with previous MI, even when baseline risk profiles are unfavorable. Although the study was conducted at a high-volume center with extensive experience in arterial grafting, the consistency of outcomes after propensity matching indicates that the benefits of MAG are not confined to a narrow, low-risk subset. Nevertheless, these favorable perioperative outcomes should be interpreted within the context of institutional expertise and may not be directly reproducible across all surgical programs.

For policymakers and health-system leaders, the study underscores the potential value of broader adoption of arterial grafting strategies. As long-term survival after CABG increasingly depends on graft durability rather than early operative risk [21], investment in training, resource allocation, and institutional pathways that facilitate MAG may yield substantial population-level benefits. Given the high prevalence of prior MI among patients referred for CABG, ensuring equitable access to arterial grafting techniques could contribute to improved long-term outcomes and reduced need for repeat revascularization. These findings also highlight the importance of incorporating conduit strategy into quality metrics and guideline development, particularly as evidence continues to accumulate in favor of multiarterial revascularization.

Despite the strengths of this study, several important questions remain unanswered and warrant further investigation. First, although long-term survival was comparable between patients with and without prior MI, the mechanisms underlying this equivalence are not fully understood. Future studies incorporating routine postoperative imaging—such as CT angiography, cardiac MRI, or stress perfusion imaging—could clarify whether graft patency, myocardial viability, or patterns of ventricular remodeling differ according to MI history and whether these factors mediate long-term outcomes.

Second, the degree, timing, and anatomical distribution of prior infarction were not captured in this dataset. It remains unclear whether patients with large anterior infarcts, multiple infarctions, or recent MI derive the same benefit from MAG as those with smaller or remote infarcts. Stratified analyses based on infarct size, transmurality, or viability could help refine patient selection and identify subgroups in whom arterial grafting is particularly advantageous—or conversely, less impactful.

Third, although this study demonstrates the safety and durability of MAG in patients with prior MI, it does not compare MAG with single arterial grafting (SAG) or hybrid revascularization strategies within this specific population. Randomized trials comparing conduit strategies in patients with prior MI are unlikely due to feasibility constraints, but large multicenter registries or collaborative propensity-matched analyses could provide valuable comparative data.

Finally, the influence of evolving surgical techniques, conduit harvesting methods, and perioperative care pathways over the 27-year study period remains an important consideration. Prospective studies with standardized operative protocols and long-term follow-up would help determine whether contemporary MAG techniques confer even greater benefit in patients with prior MI. As arterial grafting continues to evolve, future research should also explore the role of emerging conduits, such as the right gastroepiploic artery or composite graft configurations, in optimizing outcomes for this high-risk population.

## 5. Conclusions

Patients with prior myocardial infarction undergoing multiple arterial coronary bypass grafting experience in-hospital outcomes and long-term survival equivalent to those without previous MI. Despite a higher baseline risk profile, prior MI was not an independent predictor of mortality. These findings suggest that, within this single-center cohort undergoing multiple arterial grafting, prior myocardial infarction was not associated with worse adjusted in-hospital outcomes or long-term survival. Although derived from a specialized high-volume single-center experience, these results provide further evidence supporting the broader applicability of MAG in appropriately selected patients.

## Figures and Tables

**Table 1:** Preoperative demographics.

	Unmatched (n = 2468)				Matched (n = 1822)			
Variable	No Previous MI (n = 1339)	Previous MI (n = 1129)	*p* Value	SMD	No Previous MI (n = 911)	Previous MI (n = 911)	*p* Value	SMD
Age	61.47 ± 9.41	60.52 ± 9.48	0.013	0.10	60.73 ± 9.5	60.64 ± 9.2	0.840	0.01
Female gender	168 (12.5)	139 (12.3)	0.860	0.01	112 (12.3)	110 (12.1)	0.886	0.01
NYHA			0.038	0.13			0.989	0.01
I	392 (29.3)	317 (28.1)			266 (29.2)	260 (28.5)		
II	652 (48.7)	515 (45.6)			429 (47.1)	433 (47.5)		
III	251 (18.7)	239 (21.2)			178 (19.5)	181 (19.9)		
IV	44 (3.3)	58 (5.1)			38 (4.2)	37 (4.1)		
Diabetes	320 (23.9)	345 (30.6)	<0.001	0.15	250 (27.4)	259 (28.4)	0.638	0.02
Hypercholesterolemia	1025 (76.5)	852 (75.5)	0.529	0.02	703 (77.2)	695 (76.3)	0.657	0.02
Hypertension	839 (62.7)	732 (64.8)	0.263	0.04	591 (64.9)	586 (64.3)	0.806	0.01
Smoking			<0.001	0.19			0.612	0.05
Never	574 (42.9)	386 (34.2)			357 (39.2)	337 (37.0)		
Ex-smoker	639 (47.7)	586 (51.9)			446 (49.0)	459 (50.4)		
Current	126 (9.4)	157 (13.9)			108 (11.9)	115 (12.6)		
Renal impairment	16 (1.2)	23 (2.0)	0.095	0.07	14 (1.5)	13 (1.4)	0.846	0.01
Dialysis	8 (0.6)	9 (0.8)	0.405	0.02	5 (0.5)	5 (0.5)	0.574	0.00
COPD/Asthma	103 (7.7)	96 (8.5)	0.461	0.03	63 (6.9)	73 (8.0)	0.373	0.04
Cerebrovascular disease	63 (4.7)	54 (4.8)	0.928	0.00	42 (4.6)	40 (4.4)	0.821	0.01
Peripheral vascular disease	95 (7.1)	84 (7.4)	0.742	0.01	63 (6.9)	69 (7.6)	0.588	0.03
AF	28 (2.1)	24 (2.1)	0.952	0.00	21 (2.3)	19 (2.1)	0.749	0.01
Extent of CAD			0.893	0.02			0.648	0.04
1	31 (2.3)	24 (2.1)			21 (2.3)	16 (1.8)		
2	148 (11.1)	120 (10.6)			100 (11.0)	95 (10.4)		
3	1160 (86.6)	985 (87.2)			790 (86.7)	800 (87.8)		
LMS disease	307 (22.9)	265 (23.5)	0.749	0.01	211 (23.2)	215 (23.6)	0.825	0.01
LVEF			<0.001	0.56			0.327	0.01
Good (>50%)	1190 (88.9)	763 (67.6)			762 (83.6)	739 (81.1)		
Fair (30–50%)	129 (9.6)	294 (26.0)			129 (14.2)	152 (16.7)		
Poor (<30%)	20 (1.5)	72 (6.4)			20 (2.2)	20 (2.2)		
Logistic EuroSCORE	3.11± 3.8	3.81± 4.9	<0.001	0.16	3.35 ± 4.4	3.6 ± 4.5	0.246	0.06

Data n (%), mean ± standard deviation. AF = atrial fibrillation; CAD = coronary artery disease; COPD = chronic obstructive pulmonary disease; LMS = left main stem; NYHA = New York Heart Association; SMD = standardized mean difference.

**Table 2:** Intraoperative characteristics.

	Unmatched (n = 2468)			Matched (n = 1822)		
Variable	No Previous MI (n = 1339)	Previous MI (n = 1129)	*p* Value	No Previous MI (n = 911)	Previous MI (n = 911)	*p* Value
OPCAB	714 (53.3)	566 (50.1)	0.114	472 (51.8)	465 (51.0)	0.743
Number of grafts	3.28 ± 0.54	3.3 ± 0.55	0.588	3.31 ± 0.56	3.29 ± 0.55	0.475
CPB time	84 [65–88]	83 [62–86]	0.812	83 [63–91]	83 [61–90]	0.824
Aortic cross clamp time	50 [38–61]	50 [37–60]	0.912	51 [37–64]	50 [36–61]	0.854
ICOR	1.18 ± 0.12	1.14 ± 0.10	0.055	1.17 ± 0.14	1.14 ± 0.13	0.674
LAD grafting	1339 (100)	1129 (100)	1.000	911 (100)	911 (100)	1.000

Data n (%), mean ± standard deviation, median [interquartile range]. CPB = cardiopulmonary bypass; ICOR = index of completeness of revascularization; LAD = left anterior descending; MI = myocardial infarction; OPCAB = off-pump coronary artery bypass.

**Table 3:** In hospital outcomes and short-term mortality.

		Unmatched (n = 2468)				Matched (n = 1822)		
Variable	No previous MI (n = 1339)	Previous MI (n = 1129)	RR (95% CI)	*p* Value	No Previous MI (n = 911)	Previous MI (n = 911)	RR (95% CI)	*p* Value
Reoperation	58 (4.3)	42 (3.7)	0.86 (0.58–1.27)	0.840	37 (4.1)	35 (3.8)	0.94 (0.60–1.48)	0.810
Tracheostomy	12 (0.9)	14 (1.2)	1.38 (0.64–2.97)	0.405	8 (0.9)	11 (1.2)	1.38 (0.56–3.41)	0.489
TIA/CVA	25 (1.9)	24 (2.1)	1.14 (0.65–1.99)	0.646	17 (1.9)	18 (2.0)	1.06 (0.55–2.04)	0.864
DSWI	18 (1.3)	15 (1.3)	0.99 (0.50–1.96)	1.000	4 (0.4)	2 (0.2)	0.50 (0.09–2.72)	0.687
RRT	23 (1.7)	29 (2.5)	1.49 (0.87–2.56)	0.149	14 (1.5)	14 (1.5)	1.00 (0.48–2.09)	1.000
Death at 30 days	20 (1.5)	29 (2.6)	1.72 (0.98–3.02)	0.056	14 (1.5)	17 (1.9)	1.21 (0.60–2.44)	0.587

CI = confidence interval; CVA = cerebrovascular accident; DSWI = deep sternal wound infection; MI = myocardial infarction; RR = risk ratio; RRT = renal replacement therapy; TIA = transient ischemic attack; values > 1 indicate higher risk in the Previous MI group.

**Table 4:** Survival rates for unmatched cohort.

Time (Years)	Survival%	95% CI
**No previous MI**		
1	97.1	96.0–97.8
5	93.9	92.4–95.1
10	88.9	86.9–90.6
15	79.2	76.3–81.8
20	65.8	62.0–69.2
25	54.9	49.6–59.9
**Previous MI**		
1	96.3	94.9–97.2
5	92.7	90.9–94.1
10	87.0	84.6–89.0
15	77.1	73.8–80.0
20	62.3	57.9–66.3
25	51.7	45.8–57.4

Log-rank *p* = 0.1057. CI = confidence interval; MI = myocardial infarction.

**Table 5:** Survival rates for matched cohort.

Time (Years)	Survival %	95% CI
**No Previous MI**		
1	97.1	95.8–98.0
5	93.9	92.1–95.4
10	89.1	86.6–91.1
15	77.9	74.2–81.1
20	64.2	59.6–68.5
25	52.4	45.8–58.5
**Previous MI**		
1	96.7	95.3–97.7
5	93.9	92.2–95.4
10	88.5	86.0–90.6
15	78.8	75.2–81.9
20	63.0	58.1–67.4
25	53.2	46.7–59.3

Log-rank *p* = 0.8142. CI = confidence interval; MI = myocardial infarction.

**Table 6:** Univariable & multivariable Cox regression for long-term mortality.

Univariable Cox Regression					Multivariable Cox Regression			
Variable	HR	95% CI Lower	95% CI Upper	*p* Value	HR	95% CI Lower	95% CI Upper	*p* Value
Age	1.063	1.053	1.074	<0.001	1.065	1.054	1.076	<0.001
Female gender	0.744	0.594	0.932	0.010	0.926	0.736	1.165	0.511
NYHA Class								
NYHA II *	1.862	1.281	2.639	0.001	1.799	1.224	2.639	0.003
NYHA III *	2.066	1.426	2.994	<0.001	2.070	1.420	3.012	<0.001
NYHA IV *	1.637	1.104	2.432	0.014	1.681	1.128	2.500	0.011
Diabetes ^	1.321	1.102	3.125	0.003	1.284	1.068	1.543	0.008
Hypercholesterolaemia	1.000	0.829	1.206	0.999				
Hypertension	0.813	0.687	0.964	0.017	0.845	0.713	1.001	0.052
Smoking	1.084	0.832	1.411	0.551				
Renal impairment	1.222	0.457	3.269	0.690				
RRT	1.309	0.421	4.073	0.642				
Pulmonary disease	0.757	0.569	1.007	0.056				
Cerebrovascular disease	0.662	0.468	0.937	0.020	0.777	0.546	1.106	0.161
Peripheral vascular disease	0.719	0.538	0.962	0.026	0.852	0.635	1.144	0.287
Preoperative AF	0.606	0.379	0.969	0.036	0.883	0.547	1.424	0.610
Extent of coronary disease	1.425	1.136	1.789	0.002	1.169	0.875	1.561	0.290
LMS disease	1.154	0.931	1.429	0.191				
Poor LVEF	1.054	0.713	1.559	0.791				
Index of revascularisation	2.918	1.737	4.901	<0.001	1.886	0.973	3.656	0.060
OPCAB ^!^	1.238	1.043	1.469	0.014	1.444	1.214	1.718	<0.001
Previous MI ^£^	0.872	0.739	1.029	0.105				

* = (vs NYHA I); ^ = (vs No diabetes); ^!^ = (vs ONCAB); ^£^ = (vs No MI); AF = atrial fibrillation; LMS = left main stem; MI = myocardial infarction; NYHA = New York Heart Association; OPCAB = off-pump coronary artery bypass; RRT = renal replacement therapy.

## Data Availability

The data supporting the findings of this study are not publicly available due to GDPR and institutional data-governance restrictions. De-identified data may be made available upon reasonable request to the corresponding author, subject to appropriate approvals and compliance with data-protection regulations.
